# Supplementary material for: 3-D cephalometry of the the orbit regarding endocrine orbitopathy, exophthalmos, and sex
Source: PLoS One. 2022 Mar 11;17(3):e0265324. doi: 10.1371/journal.pone.0265324 (PMC8916626; doi:10.1371/journal.pone.0265324)
Supplement: Attachment — Submitted filename: PONE-D-21-33222.pdf [file pone.0265324.s002.pdf]

# PLOS ONE

## 3-D cephalometry of the the orbit in patients with and without endocrine orbitopathy (EO)

--Manuscript Draft--

| | |
|---|---|
| **Manuscript Number:** | PONE-D-21-33222 |
| **Article Type:** | Research Article |
| **Full Title:** | 3-D cephalometry of the the orbit in patients with and without endocrine orbitopathy (EO) |
| **Short Title:** | Orbital cephalometry in endocrine orbitopathy, exophthalmos, and sex |
| **Corresponding Author:** | Konstantin Volker Hierl<br>Universität Leipzig Medizinische Fakultät: Universitat Leipzig Medizinische Fakultat<br>Leipzig, GERMANY |
| **Keywords:** | endocrine orbitopathy; 3-D cephalometry; orbit |
| **Abstract:** | Purpose<br>This study aims at evaluating the orbital anatomy of patients concerning the relevance of orbital anatomy in the etiology of EO (endocrine orbitopathy) and exophthalmos utilizing a novel approach regarding three-dimensional measurements. Furthermore, sexual dimorphism in orbital anatomy was analyzed.<br>Methods<br>Orbital anatomy of 123 Caucasian patients (52 with EO, 71 without) was examined using computed tomographic data and 3-D cephalometry.<br>Results<br>Orbital anatomy was highly symmetrical with a mean side difference of 0.3 mm for distances and 0.6° for angles. There was a small albeit statistically significant difference in 13 out of 155 distances in women and 1 in men concerning patients with and without EO. 2 out of 12 angles showed a statistically significant difference between female patients with and without EO. Regarding sex, statistically significant differences occurred in 39 distances, orbit volume, orbit surface, and 2 angles. On average, measurements were larger in men. Concerning globe position within the orbit, larger distances to the orbital apex correlated with larger orbital dimensions, whereas the sagittal position of the orbital rim defined Hertel values.<br>Conclusion<br>There appears to be little difference in orbital anatomy between patients with and without EO. Concerning sex, orbital anatomy differs significantly with men presenting larger orbital dimensions. Regarding clinically measured exophthalmos, orbital aperture anatomy is an important factor which has to be considered in distinguishing between true exophthalmos with a larger distance between globe and orbital apex and pseudoexophthalmos were the orbital rim is retruded.<br>Orbital anatomy is likely to be of minor relevance in the etiology of EO but may influence therapy regarding timing and surgical procedures as orbital rim anatomy affects exophthalmos. Sex-based differences were confirmed. |
| **Order of Authors:** | Konstantin Volker Hierl |
| | Matthias Krause, MD DDS |
| | Daniel Kruber, ScD Dipl.-Inf |
| | Ina Sterker, MD PhD |
| **Additional Information:** | |
| **Question** | **Response** |
| **Financial Disclosure**<br><br>Enter a financial disclosure statement that describes the sources of funding for the work included in this submission. Review | The authors received no specific funding for this work.<br><br>The development of the 3-D analysis software (FAT) is partially funded by German Federal Ministry of Economics and Technology ZIM KF2036708SS0.<br>The funders had no role in study design, data collection and analysis, decision to |

Powered by Editorial Manager® and ProduXion Manager® from Aries Systems Corporation

| | |
|---|---|
| the submission guidelines for detailed requirements. View published research articles from *PLOS ONE* for specific examples.<br><br>This statement is required for submission and **will appear in the published article** if the submission is accepted. Please make sure it is accurate.<br><br>**Unfunded studies**<br>Enter: *The author(s) received no specific funding for this work.*<br><br>**Funded studies**<br>Enter a statement with the following details:<br>• Initials of the authors who received each award<br>• Grant numbers awarded to each author<br>• The full name of each funder<br>• URL of each funder website<br>• Did the sponsors or funders play any role in the study design, data collection and analysis, decision to publish, or preparation of the manuscript?<br>• **NO** - Include this sentence at the end of your statement: *The funders had no role in study design, data collection and analysis, decision to publish, or preparation of the manuscript.*<br>• **YES** - Specify the role(s) played.<br><br>* typeset | publish, or preparation of the manuscript. |
| **Competing Interests**<br><br>Use the instructions below to enter a competing interest statement for this submission. On behalf of all authors, disclose any competing interests that could be perceived to bias this work—acknowledging all financial support and any other relevant financial or non-financial competing interests.<br><br>This statement is required for submission and **will appear in the published article** if the submission is accepted. Please make sure it is accurate and that any funding sources listed in your Funding Information later in the submission form are also | The authors have declared that no competing interests exist. |

| | |
|---|---|
| declared in your Financial Disclosure statement.<br><br>View published research articles from *PLOS ONE* for specific examples.<br><br>**NO authors have competing interests**<br><br>Enter: *The authors have declared that no competing interests exist.*<br><br>**Authors with competing interests**<br><br>Enter competing interest details beginning with this statement:<br><br>*I have read the journal's policy and the authors of this manuscript have the following competing interests: [insert competing interests here]*<br><br>* typeset | |
| **Ethics Statement**<br><br>Enter an ethics statement for this submission. This statement is required if the study involved:<br><br>• Human participants<br>• Human specimens or tissue<br>• Vertebrate animals or cephalopods<br>• Vertebrate embryos or tissues<br>• Field research<br><br>Write "N/A" if the submission does not require an ethics statement.<br><br>General guidance is provided below. Consult the submission guidelines for detailed instructions. **Make sure that all information entered here is included in the Methods section of the manuscript.** | Our study was approved by the Leipzig University ethics commission (No. 285-14-25082014) and performed in accordance with the Declaration of Helsinki.<br><br>The data were analyzed anonymously. |

| | |
|---|---|
| **Format for specific study types**<br><br>**Human Subject Research (involving human participants and/or tissue)**<br>• Give the name of the institutional review board or ethics committee that approved the study<br>• Include the approval number and/or a statement indicating approval of this research<br>• Indicate the form of consent obtained (written/oral) or the reason that consent was not obtained (e.g. the data were analyzed anonymously)<br><br>**Animal Research (involving vertebrate animals, embryos or tissues)**<br>• Provide the name of the Institutional Animal Care and Use Committee (IACUC) or other relevant ethics board that reviewed the study protocol, and indicate whether they approved this research or granted a formal waiver of ethical approval<br>• Include an approval number if one was obtained<br>• If the study involved *non-human primates*, add *additional details* about animal welfare and steps taken to ameliorate suffering<br>• If anesthesia, euthanasia, or any kind of animal sacrifice is part of the study, include briefly which substances and/or methods were applied<br><br>**Field Research**<br><br>Include the following details if this study involves the collection of plant, animal, or other materials from a natural setting:<br>• Field permit number<br>• Name of the institution or relevant body that granted permission | |
| **Data Availability**<br><br>Authors are required to make all data underlying the findings described fully available, without restriction, and from the time of publication. PLOS allows rare exceptions to address legal and ethical concerns. See the PLOS Data Policy and FAQ for detailed information. | Yes - all data are fully available without restriction |

*Powered by Editorial Manager® and ProduXion Manager® from Aries Systems Corporation*

| | |
|---|---|
| A Data Availability Statement describing where the data can be found is required at submission. Your answers to this question constitute the Data Availability Statement and **will be published in the article**, if accepted.<br><br>**Important:** Stating 'data available on request from the author' is not sufficient. If your data are only available upon request, select 'No' for the first question and explain your exceptional situation in the text box.<br><br>Do the authors confirm that all data underlying the findings described in their manuscript are fully available without restriction? | |
| **Describe where the data may be found in full sentences. If you are copying our sample text, replace any instances of XXX with the appropriate details.**<br><br>• If the data are **held or will be held in a public repository**, include URLs, accession numbers or DOIs. If this information will only be available after acceptance, indicate this by ticking the box below. For example: *All XXX files are available from the XXX database (accession number(s) XXX, XXX.).*<br>• If the data are all contained **within the manuscript and/or Supporting Information files**, enter the following: *All relevant data are within the manuscript and its Supporting Information files.*<br>• If neither of these applies but you are able to provide **details of access elsewhere**, with or without limitations, please do so. For example:<br><br>*Data cannot be shared publicly because of [XXX]. Data are available from the XXX Institutional Data Access / Ethics Committee (contact via XXX) for researchers who meet the criteria for access to confidential data.*<br><br>*The data underlying the results presented in the study are available from (include the name of the third party* | All relevant data are within the manuscript and its Supporting Information files. |

| | |
|---|---|
| *and contact information or URL).*<br>• This text is appropriate if the data are owned by a third party and authors do not have permission to share the data.<br><br>* typeset | |
| Additional data availability information: | |

Manuscript

Click here to access/download;Manuscript;Manuscript - 3-D cephalometry of the the orbit.docx.docx

# 3-D cephalometry of the the orbit regarding endocrine orbitopathy (EO), exophthalmos, and sex

Short title:

## Orbital cephalometry in endocrine orbitopathy, exophthalmos, and sex

**Konstantin Volker Hierl[1*], Matthias Krause MD DDS[2], Daniel Kruber ScD Dipl.-Inf[3], Ina Sterker MD PhD[1]**

[1] Department of Ophthalmology, Leipzig University. Liebigstr. 12, 04103 Leipzig, Germany

[2] Department of Oral & Maxillofacial Plastic Surgery, Leipzig University. Liebigstr. 12, 04103 Leipzig, Germany

[3] Department of Informatics and Media, Leipzig University of Applied Sciences, Karl-Liebknecht-Str. 145, 04277 Leipzig, Germany

* Corresponding author

E-mail: konstantin.hierl@gmx.de

Email-addresses:
konstantin.hierl@gmx.de
KrauseMa@medizin.uni-leipzig.de
danielkruber@gmx.de
Ina.Sterker@medizin.uni-leipzig.de

// Abstract

### Purpose

This study aims at evaluating the orbital anatomy of patients concerning the relevance of orbital anatomy in the etiology of EO (endocrine orbitopathy) and exophthalmos utilizing a novel approach regarding three-dimensional measurements. Furthermore, sexual dimorphism in orbital anatomy was analyzed.

### Methods

Orbital anatomy of 123 Caucasian patients (52 with EO, 71 without) was examined using computed tomographic data and 3-D cephalometry.

### Results

Orbital anatomy was highly symmetrical with a mean side difference of 0.3 mm for distances and 0.6° for angles. There was a small albeit statistically significant difference in 13 out of 155 distances in women and 1 in men concerning patients with and without EO. 2 out of 12 angles showed a statistically significant difference between female patients with and without EO. Regarding sex, statistically significant differences occurred in 39 distances, orbit volume, orbit surface, and 2 angles. On average, measurements were larger in men. Concerning globe position within the orbit, larger distances to the orbital apex correlated with larger orbital dimensions, whereas the sagittal position of the orbital rim defined Hertel values.

### Conclusion

There appears to be little difference in orbital anatomy between patients with and without EO. Concerning sex, orbital anatomy differs significantly with men presenting larger orbital dimensions. Regarding clinically measured exophthalmos, orbital aperture anatomy is an important factor which has to be considered in distinguishing between true exophthalmos with a larger distance between globe and orbital apex and pseudoexophthalmos were the

orbital rim is retruded.

Orbital anatomy is likely to be of minor relevance in the etiology of EO but may influence therapy regarding timing and surgical procedures as orbital rim anatomy affects exophthalmos. Sex-based differences were confirmed.

## Introduction

Endocrine orbitopathy (EO) is an inflammatory autoimmune disease affecting the orbit occurring in 16/100,000 women and 2.9/100,000 men per year with an onset between 30 and 60 years [1]. EO is typically associated with Graves' disease and one of its most relevant extrathyreoidal manifestations, but may also occur in association with other diseases of the thyroid [2].

Characteristic symptoms in EO include exophthalmos, upper eyelid retraction, chemosis, conjunctival injection and diplopia. Loss of vision due to optic neuropathy is a feared complication [3].

The most important aspects in the management of EO are the restoration and maintainance of euthyreoidism as well as immunosuppressive therapy [4]. Rehabilitative surgery is an option in stable and inactive EO as well as in vision-threatening EO [4].

Many surgical methods for decompression (i.e. resection) of one to four orbital walls with or without orbital fat removal have been established since the first description of orbital decompression by Dollinger in 1911 [5, 6, 7]. All surgical therapies necessitate the knowledge of orbital anatomy and the anatomy in EO for both the planning of surgery as well as the evaluation of surgical success.

The purpose of our study was to evaluate orbital anatomy in patients with and without EO investigating the relevance of orbital anatomy in the etiology of EO and exophthalmos – one of its major clinical features – using a new approach of three-dimensional cephalometric

measurement. In previous investigations, Baujat et al. and Rajabi et al. [8, 9] did not find such significant anatomical differences besides slight differences in the lateral orbital angle (angle between the midsagittal plane and the lateral orbital wall) or interorbital distance. These studies, however, were limited by the use of axial CT slices for 2-D measurements instead of 3-D cephalometry [8, 9], by comparing two patient groups with exophthalmos (EO and non-EO) [8] or small patient groups and only non-EO patients [9] and their limited number of parameters. Furthermore, the influence of sex was not evaluated.

As EO occurs more often in women than in men, we also studied anatomical differences between men and women. While such differences have been described before [10, 11], we included more anatomical parameters than previous observations and utilized the possibilites of 3-D cephalometry.

Furthermore we wanted to evaluate the influence of anatomical traits on the extent of exophthalmos in EO and non-EO patients as these anatomical factors could contribute to an appearance which could lead patients to seek treatment. Regarding surgery in EO patients, these potential traits could be useful in deciding on appropriate surgical procedures.

Finally we wanted to evaluate the symmetry of orbital anatomy besides overall orbital shape as methods based on mirroring and using the unaffected side as a model [12] are important in surgical reconstruction concerning orbital trauma and disease.

## Materials and methods

We evaluated computed tomographic data of 123 adult Caucasian patients with a mean age of 44.0 ±15.7 years (range 18 – 84 years). Of those patients, 52 (42.3%) had known EO and

were scheduled for decompression surgery. 71 patients (57.7%) had no known pathology of the orbit (e.g. trauma, tumors, deformities) and constituted our reference group. Besides the absence of other pathologies of the orbit, the CTs had to have a slice thickness of not more than 1.5 mm with contiguous slicing to be included in our study.

Out of the 52 patients with known EO, 35 (67.3%) were female and 17 (32.7%) male. Their mean age was 49.1 ±10.4 years (range 28 – 76 years). In contrast, our reference group of 71 patients consisted of 46 (64.8%) male and 25 (35.2%) female patients with a mean age of 40.2 ± 17.8 years (range 18 – 84 years) without known orbital pathologies.

To measure the orbital volume and surface, we imported the CT data into iPlan Brainlab (Brainlab AG, Feldkirchen, Germany) software and used automatic segmentation with subsequent manual adjustments [13]. Subsequently we imported the resulting STL (Standard Triangulation Language) files into FAT (Facial Analysis Tool) software to measure surface and volume. All 3-D measurements were made in FAT software [14, 15]. We developed a cephalometric analysis using 56 landmarks (5 unilateral landmarks, 21 bilateral landmarks, 3 unilateral constructed landmarks, 3 bilateral; Tab. 1, Fig. 1), 14 planes, 20 angles and measured 155 distances (70 bilateral, 15 unilateral) (Tab. 2, Fig. 2). Landmarks were placed either directly on the 3-D object or by using crosshairs in landmarks not associated with bone. Crosshairs could be utilized in the 3-D reconstruction or on three multiplanar reformatted (MPR) image planes which could be overlaid with the 3-D object (which then could be faded out). Thus the center of the optic canal was marked on the 3-D display, whereas *Sella* point was defined on the overlaid MPRs in the 3-D window, while the location of the landmarks was additionally shown on each plane in a separate window. The cephalometric analysis was created by using a construction matrix using landmarks to define lines, planes, distances, and angles [14]. The great advantage of this approach is that in having landmark data sets and an analysis matrix, modifications within the landmark data

or analysis can be performed without repeating the whole data acquisition. The analysis results were exported into an excel spreadsheet and then imported into the statistical analysis software.

Table 1: 3-D landmarks placed in FAT and their respective definitions, constructed planes and angles.

Tab. 1

| | | |
|---|---|---|
| **Anatomical landmarks** | | |
| Apex orbitae (bilateral) | AO | central point of optic canal at the border of the orbit |
| Apex orbitae fissure (bilateral) | Aof | most medial caudal point of superior orbital fissure |
| A-Point | A | most retral point of the curvature of the upper alveolus (in MSP plane) |
| Basion | Ba | most anterior point of foramen magnum in midsagittal plane |
| Dakryon (bilateral) | Da | contact point of maxilla,frontal bone, and lacrimal bone |
| | | (contact anterior lacrimal crest and maxillofrontal suture) |
| Frontomalar suture (bilateral) | Fms | medial-anterior point of frontozygomatic suture |
| Midpoint lateral orbital rim (bilat.) | Mlo | midpoint of lateral orbital rim |
| Infraorbitale (bilateral) | In | most superior point of infraorbital foramen |
| Infraorbital midpoint (bilateral) | Inf | midpoint of intraorbital rim |
| Infraorbital rim lateral (bilateral) | Il | most lateral point of infraorbital rim |
| Infraorbital rim medial (bilateral) | Im | most medial point of infraorbital rim |
| Orbitale (bilateral) | Or | most caudal point on infraorbital rim |
| Nasion (bone) | N | midpoint of fronto-nasal suture |
| Porion (bilateral) | Po | most superior point of the external auditory meatus |
| Sella | S | center of sella turcica |
| Anterior sphenoid trigone (bilat.) | Ast | anterior border of the sphenoid trigone (level of the midpoint of the lateral orbital wall) |
| Posterior sphenoid trigone (bilat.) | Pst | posterior border of the sphenoid trigone (level of the midpoint of the lateral orbital wall) |
| Supraorbitale (soft tissue) bilat.) | So | point overlying bony Suparorbitale |
| Supraorbital foramen (bilateral) | SupF | central-caudal point of supraorbital foramen |
| Supraorbital midpoint (bilateral) | SupM | midpoint of supraorbital rim |
| Globe anterior (bilateral) | Ga | most anterior point of globe diameter |
| Globe posterior (bilateral) | Gp | most posterior point of globe diameter |
| Orbitale soft tissue (bilateral) | Or | soft tissue point overlying bony orbitale |
| Exokanthion (bilateral) | Exo | most lateral point of palpebral fissure |
| Endokanthion (bilateral) | Endo | most medial point of palpebral fissure |
| Nasion soft tissue | N | point overlying bony Nasion |
| **Constructed landmarks** | | |
| Midaperture (bilateral) | mA | midpoint of orbital aperture; lines connecting (Inf-SupM) and (Mlo-Da) |
| Midpoint globe (bilateral) | mG | midpoint of globe |
| Midporion | mPo | midpoint between left-right Porion |
| Midpoint Mlo | mMlo | midpoint left-right midpoint lateral orbital rim |
| Midapex | mApex | midpoint Aof bilateral |
| Midpoint lid aperture (bilateral) | mL | midpont-exo-endo |
| **Planes** | | |

| | | |
|---|---|---|
| Frankfurt Horizontal | FH | Porion right- Porion left - Infraorbitale left |
| Midsagittal plane | MSP | perpendicular to FH through N and mPo |
| Coronar plane | Cor | perpendicular to FH and MSP, through right Porion |
| parallel planes to Cor | Corpara-… | through landmarks Da, SupM, Ga, Mlo, AO, Aof, Ma, Inf, S, Ba, Fms |
| parallel planes to FH | FHpara… | through landmarks Mlo, … |
| **Angles** | | |
| SNA° | internal angle between S, N, and A landmarks | |
| BaSN° | internal angle between Ba, S, and N landmarks | |
| latorbangle ° | internal angle between left-right Fms and S | |
| medorbangle° | internal angle between left-right Da and S | |
| latorb-MSP° (bilateral) | internal angle between Mlo-S and MSP | |
| latorb-MSP2° (bilateral) | internal angle between Mlo-AO and MSP | |
| Horizontal orbit angle° (bilateral) | internal angle between lateral and medial orbital walls (Mlo-AO and Da-AO) | |
| Vertical orbital angle° (bilateral) | internal angle between SupM-AoF and Inf-AoF | |
| Infraorb slope° (bilateral) | internal angle Inf-AoF and FH | |
| lateral orbital slope° (bilateral) | internal angle between line left-right Mlo and Da (left/right)-Mlo (left/right) | |
| Orbithorizontal-Cor° (bilateral) | internal angle between Da-Mlo and Cor | |
| Globe axis° (bilateral) | internal angle Ga-Gp and MSP | |

Fig. 1: Visualization of 3-D landmarks on a 3-D reconstruction of the skull made in FAT.

Table 2: Distances measured in the study and their definitions.

| **Distances** | | | | | |
|---|---|---|---|---|---|
| posterior interorbital width | distance AO bilateral | bony Hertel FMS (bilateral) | Fms-Corpara-Ga | GlobeparalCor1 (bilateral) | Ga-Corpara-Mlo |
| Interorbitwidth posterior | distance Aof bilateral | Apex orbitae-Cor plane (bilateral) | AO-Cor | Endokantion RL | endo-endo |
| anterior interorbital width | distance Da bilateral | infraorbital depth (bilateral) | Inf-Cor | Exokantion RL | exo-exo |
| InterFrontomalarsut Distance | distance Fms bilateral | orbitdepht Aof (bilateral) | Aof-mA | Globe position anterior (bilateral) | Ga-Corpara-mMlo |
| InterMidpoint latorb Distance | distance Mlo bilateral | orbitdepth anterior (bilateral) | Da-Corpara-mMlo | Globe lateral position (bilateral) | mG-MSP |
| Orbital width (bilateral) | Da-Mlo | infraorbit-cor plane Supraorb (bilat.) | Inf-Corpara-SupM | Globe-Supraorbital plane (bilat.) | Ga-Corpara-So |
| Orbital height (bilateral) | Or-SupF | Mlo-coronal plane Da (bilateral) | Mlo-Corpara-Da | Globe-Or-plane (bilateral) | Ga-Corpara-Or |
| Orbit height 2 (bilateral) | Inf-SupM | Inf-coronal plane Da (bilateral) | Inf-Corpara-Da | Globe Protrusion (bilateral) | Ga-mA |
| orbitlength med (bilateral) | Da-AO | Sphenoidtrigone depth (bilateral) | Ast-Pst | midglobe-coronal plane Da (bilateral) | mG-Corpara-Da |
| orbitlength lateral (bilateral) | Mlo-AO | lateral orbit-Sphenoid anterior (bilat.) | Mlo-Ast | anterior globe coronal plane Da (bilat.) | Ga-Corpara-Da |
| orbitlenght superior (bilateral) | Aof-SupM | lateral orbit-Sphenoid posterior (bilat.) | Mlo-Pst | anterior globe coronal plane Mlo (bilat.) | Ga-Corpara-Mlo |
| orbitlenght inferior (bilateral) | Aof-Inf | Exokant-coronal plane (bilateral) | exo-Cor | midglobe-coronal plane Mlo (bilateral) | mG-Corpara-Mlo |
| orbitlenght lateral (Aof) bilateral) | Aof-Mlo | Endokantion-coronal plane (bilateral) | endo-Cor | Lid aperture (bilateral) | exo-endo |
| Fms-Cor (bilateral) | Fms-Cor | A Point-Midapertureplane (bilateral) | A-Corpara-mA | Globe coronar plane-N (bilat.) | Ga-Corpara-N |
| Mlo-Cor (bilateral) | Mlo-Cor | Supraorbital soft tissue-Cor plane (bilat.) | So-Cor | Globe-coronar plane-exo (bilat.) | Ga-Corpara-exo |
| Mlo-Midsag plane (bilateral) | Mlo-MSP | Orbitale soft tissue-Cor plane (bilat.) | Or-Cor | Globe position horizontal (bilateral) | mG-MSP |
| Fms-Midsag plane (bilateral) | Fms-MSP | Globe distance | distance Ga bilateral | Globe position vertical (bilateral) | mG-Fhpara-mA |
| Orbit depth (bilateral) | mA-AO | Globe diameter (bilateral) | Ga-Gp | midlat. orbit-cor. plane Supraorbit (bilat.) | Mlo-Corpara-SupM |
| Orbitdepth3 (bilateral) | mG-Corpara-mApex | lid midpoint-Apex orbitae (bilateral) | mL-AO | Dakryon-coronal plane Supraorbit (bilat.) | Da-Corpara-SupM |
| Orbitale-Cor (bilateral) | Or-Cor | midpoint Globe Cor plane-S (bilateral) | mG-Corpara-S | infraorbit-coronal plane Supraorbit (bilat.) | Inf-Corpara-SupM |
| Dakryon-Cor (bilateral) | Da-Cor | Globe-coronar plane-Endok (bilateral) | Ga-Corpara-endo | mid-lateral orbit-coronal plane Da (bilat.) | Mlo-Corpara-Da |
| Supraorbital foramen-cor. plane (bilat.) | Supf-Cor | MidGlobe-Apex orbitae (bilat.) | mG-AO | mid-lateral orbit-coronal plane Ao (bilat.) | Mlo-Corpara-AO |
| Nasion soft tissue-coronal plane | n-Cor | Globe posterior Apex (bilateral) | Gp-AO | Dakryon-coronal plane Apex orbitae (bilat.) | Da-Corpara-AO |
| Nasion-coronal plane | N-Cor | bony Globe protrusion (bilateral) | mA-Ga | supraorbit-cor. plane Apex orbitae (bilat. | SupM-Corpara-AO |
| mid Orbit-Sella plane | mMlo-Corpara-S | bony Gobe post protrusion (bilateral) | mA-Gp | infraorbit-coronal plane AO (bilateral) | Inf-Corpara-AO |
| mid Orbit-Basion plane | mMlo-Corpara-Ba | bony Globe mid protrusion (bilateral) | mA-mG | midpoint Mlo-coronal plane S | mMlo-Corpara-S |
| bony Hertel (bilateral) | Mlo-CorparaGa | Globe position sagittal (bilateral) | Aof-Ga | midpoint Mlo-coronal plane Ba | mMlo-Corpara-Ba |

| | | | | | |
|---|---|---|---|---|---|
| bony Hertel midaperture (bilateral) | Mlo-Corpara-mA | Globe-coronal plane (bilat.) | Ga-Cor | Midaperture-Cor (bilateral) | mA-Cor |

Fig. 2: Visualization of distances shown in Tab. 2.

Statistical analysis was then performed with Winmedas (Fa. Christian Grund, Margetshoechheim, Germany) software. We assessed the anatomical data of patients with and without known EO as well as male and female patients in unconnected t-tests and the symmetry between left and right orbit in connected t-tests. Correlations were measured using Spearman's rank correlation test.

Results with $p \leq 0.05$ were regarded as statistically significant.

This retrospective study was approved by the local ethics commission (No. 285-14-25082014) and followed the Declaration of Helsinki on medical protocols and ethics.

# Results

Globally, the orbital anatomy measures proved to be highly symmetrical with an average side difference in distances of 0.3 mm and no mean difference in any distance greater than 0.6 mm. The average side difference regarding angles was 0.6° with no mean side difference being greater than 1.7°. This observation was valid in all groups.

The comparison of the orbital anatomy in patients with and without EO showed statistically significant differences in 13 out of 155 distances (Tab. 3) in women and in one distance in men. Women with EO had a larger orbital volume and concomitantly orbital surface area, which was related to a larger orbital height, width, and length. The *medorbangle* (measured between left and right Dakryon and Sella) was smaller in women with EO which originated from a smaller anterior interorbital width (distance left – right *Dakryon*). Women with EO presented a larger *horizontal angle* (the angle between medial and lateral orbital walls). Furthermore, *Orbitale* (most caudal landmark on the infraorbital rim) was positioned more anterior in female EO patients. Albeit statistically different, those differences were relatively small.

While these parameters showed no statistically significant differences in men, the position of the anterior border of the *sphenoid trigone* was more anterior in men with EO (the only specifically male difference), while the length of the sphenoid trigone was increased in women with EO. Without discerning sex, only *orbital height*, *sphenoid trigone length*, and *horizontal orbital angle* were increased in EO, while the *posterior interorbital width* (measured at *Apex orbitae*) was decreased.

Table 3: Comparison of EO versus nonEO CT scans

| | EO | nEO | | EO male | nEO male | | EO female | nEO female | |
|---|---|---|---|---|---|---|---|---|---|
| | Mean ± SD | Mean ± SD | p | Mean ± SD | Mean ± SD | p(t) | Mean ± SD | Mean ± SD | p |
| **Volume/surface** | | | | | | | | | |
| orbit volume | 29.9 ± 3.2 | 29.4 ± 2.7 | n.s. | 30.9 ± 3.4 | 30.4 ± 2.2 | n.s. | 29.3 ±3 | 27.5 ± 2.4 | 0.017 |
| orbit surface | 56 ± 3.8 | 55 ± 3.3 | n.s. | 57.3 ± 3.9 | 56.5 ± 2.7 | n.s. | 55.3 ± 3.7 | 52.7 ± 2.9 | 0.005 |
| **Distances** | | | | | | | | | |
| orbit width | 42.1 ± 1.9 | 41.5 ± 2 | n.s. | 42.5 ± 1.5 | 42 ± 1.7 | n.s. | 41.8 ± 2.1 | 40.4 ± 2.3 | 0.020 |
| orbit height | 42.7 ± 2.2 | 41.4 ± 2.5 | 0.002 | 42.5 ± 1.9 | 41.5 ± 2.6 | n.s. | 42.8 ± 2.4 | 41.2 ± 2.3 | 0.009 |
| length lateral orbit (AO) | 47.8 ± 2.1 | 47.2 ± 2.4 | n.s. | 48.6 ± 2.3 | 47.8 ± 2.4 | n.s. | 47.4 ± 1.9 | 45.9 ± 1.9 | 0.004 |
| orbit depth (mA-AO) | 44.8 ± 2.4 | 44.3 ± 2.6 | n.s. | 45.6 ± 2.4 | 45.1 ± 2.6 | n.s. | 44.4 ± 2.3 | 43 ± 2.1 | 0.016 |
| Sphenoid trigone (Ast-Pst) | 10.3 ± 2.6 | 8.8 ± 2.8 | 0.003 | 10.2 ± 2.5 | 8.9 ± 2.9 | n.s. | 10.4 ± 2.7 | 8.8 ± 2.6 | 0.021 |
| Mlo-Ast | 17.9 ± 2.4 | 19.6 ± 2.5 | < 0.001 | 18.2 ± 2.3 | 20.2 ± 2.5 | 0.006 | 17.8 ± 2.5 | 18.5 ± 2.1 | n.s. |
| Mlo-Pst | 27.6 ± 3.2 | 27.5 ± 3.5 | n.s. | 27.7 ± 2.8 | 27.9 ± 3.7 | n.s. | 27.5 ± 3.4 | 26.6 ± 3.1 | n.s. |
| Mlo-cor1Aof | 34.5 ± 2.4 | 34.5 ± 2.6 | n.s. | 35 ±2.4 | 35.2 ±2.6 | n.s. | 34.3 ± 2.3 | 33 ± 2 | 0.040 |
| **Angles** | | | | | | | | | |
| medorb angle | 19.8 ± 2.6 | 20.3 ± 2.8 | n.s. | 20.6 ± 2.1 | 19.7 ± 2.6 | n.s. | 19.4 ± 2.7 | 21.3 ± 3 | 0.009 |
| horizontal orbit angle | 51.5 ± 3.9 | 50.2 ± 2.7 | 0.043 | 51.3 ± 4.2 | 50.6 ± 2.5 | n.s. | 51.5 ± 3.8 | 49.6 ± 3 | 0.036 |
| **orbit-skull base** | | | | | | | | | |
| posterior interorbital width | 30.3 ± 3.2 | 31.5 ± 3.4 | 0.049 | 31.6 ± 3.5 | 31.9 ± 3.1 | n.s. | 29.7 ± 2.9 | 30.9 ± 3.9 | 0.18 |
| anterior interorbital width | 20.6 ± 2.7 | 21.3 ± 2.7 | n.s. | 21.9 ± 2.3 | 21.1 ± 2.7 | n.s. | 19.9 ± 2.6 | 21.6 ± 2.6 | 0.016 |
| or-cor1 | 90.3 ± 4.3 | 88.6 ± 5.9 | n.s. | 91.7 ± 4.1 | 90 ± 5.7 | n.s. | 89.6 ± 4.2 | 85.9 ± 5.3 | 0.004 |

Concerning sex, distinct differences were found. Tab. 4 shows these which were seen in both EO and non-EO patients regarding hard tissue measurements. Thus values for all patients are stated for bony measurements, only concerning soft tissue values (which would be influenced by EO), the results of the non-EO group are given. Men showed a significantly larger orbital volume and surface area which was related to a larger orbital width, depth, and longer orbital walls with a more anterior positioned lateral orbital rim midpoint. Men presented a more anterior supraorbital rim (in relation to the lateral orbital wall and *Dakryon*), and a more anterior placed orbit in relation to the middle cranial fossa. Regarding soft tissue differences in the non-EO group, men presented more protrusion of the supraorbital rim and *Exo-Endokanthion* in relation to the coronal plane. While these differences ranged from 1 – 2 mm for most parameters, supraorbital rim protrusion was marked with an average difference of 13 mm. Regarding angle measurements, only *medorbangl*e was different, being larger in women.

Table 4: Sex differences. Bony measurements include all CT scans, whereas soft tissue variables were made for the nonEO group only.

| | Mean±SD | Mean±SD | p | | Mean±SD | Mean±SD | p |
|---|---|---|---|---|---|---|---|
| | male | female | | | male | female | |
| **volume/surface** | | | | **skull base middle cranial fossa - orbit** | | | |
| orbit volume | 30.6 ± 2.6 | 28.6 ± 2.9 | < 0.001 | mMlo-CorparaS | 47.8 ± 3.1 | 44.9 ± 2.5 | < 0.001 |
| orbit surface | 56.7 ± 3 | 54.3 ± 3.6 | < 0.001 | mMlo-CorparaBa | 68.9 ± 4.6 | 65.2 ± 3.4 | < 0.001 |
| **distances** | | | | posterior interorbital width | 31.8 ± 3.1 | 30.2 ± 3.4 | 0.007 |
| orbit width | 42.2 ± 1.6 | 41.2 ± 2.2 | 0.012 | AO-Cor | 38.2 ± 3.7 | 36.8 ± 3.6 | 0.048 |
| inter-midlatorb (Mlo-Mlo) | 98.8±4.1 | 96.9±3.5 | 0.044 | SupM-Cor | 87 ± 4.9 | 82.9 ± 4.5 | < 0.001 |
| orbit length med | 44.7 ± 3 | 43.4 ± 2.7 | 0.014 | Or-Cor | 77.1 ± 4.3 | 74.2 ± 4.1 | < 0.001 |
| orbit length lat | 48.1 ± 2.3 | 46.8 ± 2 | 0.003 | Fms-Cor | 72.2 ± 4 | 69.4 ± 3.6 | < 0.001 |
| orbit depth | 45.2 ± 2.6 | 43.8 ± 2.3 | 0.003 | Mlo-Cor | 70.2 ± 4 | 67.6 ± 3.4 | < 0.001 |
| orbit length sup | 54.9 ± 2.7 | 53.1 ± 2.8 | < 0.001 | Fms-MSP | 50.4 ± 2.3 | 49 ± 1.6 | < 0.001 |
| orbit length inf | 50 ± 2.6 | 48.3 ± 2.8 | 0.001 | mA-Cor | 76.3 ± 4.1 | 73.7 ± 3.8 | < 0.001 |
| orbit lenght lat (AoF) | 48.1 ± 2.3 | 46.6 ± 2.3 | 0.001 | Da-Cor | 82.5 ± 4.7 | 79.9 ± 4.5 | 0.002 |
| orbit depth | 42.7 ± 2.4 | 41.3 ± 2.5 | 0.001 | or-Cor | 90.5 ± 5.4 | 88.1 ± 5 | 0.011 |
| Mlo-Ast | 19.6 ± 2.6 | 18 ± 2.4 | < 0.001 | **Soft tissue anatomy; only non-EO** | | | |
| Mlo-CorparaSupM | 16.9 ± 2.9 | 15.5 ± 2.5 | 0.011 | So-Cor | 95.8 ± 5.4 | 90.4 ± 5.8 | < 0.001 |
| Da-CorparaSupM | 47.4 ± 2.8 | 33.9 ± 2.1 | 0.003 | exo-Cor | 77.1 ± 4.7 | 74.3 ± 4.2 | 0.016 |
| Mlo-CorparaAof | 35.2 ± 2.5 | 33.7 ± 2.3 | 0.001 | endo-Cor | 85.4 ± 5 | 82.3 ± 4.8 | 0.013 |
| Da-CorparaAof | 44.3 ± 3 | 43 ± 2.7 | 0.014 | **Bulbus position** | | | |
| **Angles** | | | | midpointGlobe-CorparaS | 41.5±3.2 | 37.8±3.5 | < 0.001 |
| medorbangle° | 19.7±2.6 | 21.3±3 | 0.02 | Orbitdepth3 | 36.9±2.8 | 34.3±2.5 | < 0.001 |
| **Proptosis** | | | | Midglobe-AO | 50.5±2.8 | 47.8±2.8 | < 0.001 |
| Ga-So | 32±2.9 | 30±3.1 | 0.009 | mG-MSP | 54.5±3 | 51.9±2.6 | < 0.001 |
| Lidmidpoint (mL)-AO | 54.8±3.2 | 52±2.5 | < 0.001 | Ga-Cor | 86.4±5 | 82.6±5 | 0.003 |
| Ga-mA | 7.5±4.2 | 9.5±4.2 | 0.007 | | | | |
| Ga-corpara-N | 12.2±4.6 | 8.7±4.6 | < 0.001 | | | | |

Analysis concerning bulbus position showed a more anterior and lateral globe position with respect to the coronal plane and *orbital apex* in men (i.e. a more anterior positioned globe in absolute measures to the coronal plane defined by *Porion* landmarks). Regarding clinically measured exophthalmos (i.e. the relative globe position to the orbital aperture), women presented a more anteriorly positioned globe regarding to *orbit midaperture* (2 mm) and *Nasion* (3.5 mm).

After investigating symmetry, EO characteristics, and sex differences, the last issue was to evaluate the influence of anatomy on orbital proptosis. As exophthalmos is a major stigma in EO, analysis of the non-EO group was of particular interest. Nevertheless, EO patients were evaluated, too, as anatomical factors could play a role in the extent of exophthalmos and thus in the decision on therapeutical measures.

The results are given in Tab. 5 & 6. First, the correlation between exophthalmos and bony distances including orbit volume and surface was evaluated. Here the distance globe-*apex orbitae* correlated with orbital dimensions. This implies that in larger orbits the distance from anterior globe to apex is also larger.

Table 5: Correlation of bony anatomic parameters (distances) to globe position values.

| | **Hertel analogue measures** | | | | **anterior Globe to AOF/mApex** | | | | **anterior Globe to MSP** | | | |
|---|---|---|---|---|---|---|---|---|---|---|---|---|
| | (Globe to Fms/Mlo/CorparaMlo/mMlo | | | | | | | | | | | |
| | EO | | nEO | | EO | | nEO | | EO | | nEO | |
| | r | p | r | p | r | p | r | p | r | p | r | p |
| **General orbit size** | | | | | | | | | | | | |
| volume | 0.31 | 0.031 | | | | | 0.41 | < 0.001 | 0.41 | 0.003 | 0.47 | < 0.001 |
| surface | 0.32 | 0.022 | 0.27 | 0.029 | | | 0.49 | < 0.001 | 0.42 | 0.002 | 0.51 | < 0.001 |
| orbit width | 0.41 | < 0.005 | 0.45 | < 0.001 | | | 0.34 | 0.004 | 0.51 | < 0.001 | 0.53 | < 0.001 |
| orbit height | 0.36 | 0.008 | | | 0.27 | 0.050 | | | 0.29 | 0.040 | 0.25 | 0.033 |
| orbit length med | 0.38 | 0.006 | 0.37 | 0.002 | | | 0.45 | < 0.001 | | | | |
| orbit length lat | 0.28 | 0.045 | | | 0.44 | 0.001 | 0.51 | < 0.001 | | | | |
| orbit depth | 0.40 | 0.003 | 0.26 | 0.027 | 0.37 | 0.007 | 0.50 | < 0.001 | | | | |
| orbitlength sup | 0.31 | 0.026 | 0.37 | 0.001 | 0.61 | < 0.001 | 0.65 | < 0.001 | | | | |
| orbit length inf | 0.46 | < 0.001 | | | 0.54 | < 0.001 | 0.64 | < 0.001 | | | | |
| orbit depth Aof | 0.28 | 0.047 | | | 0.55 | < 0.001 | 0.71 | < 0.001 | | | | |
| infraorb depth | 0.42 | 0.002 | 0.34 | 0.004 | | | 0.46 | < 0.001 | 0.28 | 0.046 | 0.33 | 0.004 |
| **Position of orbital rim** | | | | | | | | | | | | |
| infmid-corSupra | 0.35 | 0.011 | 0.29 | 0.016 | | | | | | | | |
| latorb-corDa | 0.48 | < 0.001 | 0.64 | < 0.001 | | | | | 0.35 | 0.010 | 0.26 | 0.026 |
| latorb-AST | | | | | 0.35 | 0.012 | 0.27 | 0.022 | | | | |
| latorb-Pst | | | -0.33 | 0.005 | | | 0.28 | 0.020 | | | | |
| latorb-corSupra | 0.48 | < 0.001 | 0.69 | < 0.001 | | | | | 0.33 | 0.016 | 0.29 | 0.013 |
| Da-corSupra | 0.31 | 0.027 | | | 0.32 | 0.019 | | | | | | |
| inforbmid-corSupra | 0.35 | 0.011 | 0.28 | 0.017 | | | | | | | | |
| latorb-corAO | | | | | 0.33 | 0.016 | 0.46 | < 0.001 | | | | |
| Da-corAO | 0.40 | 0.003 | 0.39 | < 0.001 | | | 0.44 | < 0.001 | | | | |
| orbit depth ant | 0.40 | 0.003 | 0.57 | < 0.001 | | | | | 0.35 | 0.012 | | |

Table 6: Correlations of angle measurements to globe position. Hertel values were defined as: *Ga-CorparaMlo* and *Fms-CorparaGa*

| | EO | nEO | | EO | | nEO | | EO | nEO | | EO | | nEO | | EO | | nEO | |
|---|---|---|---|---|---|---|---|---|---|---|---|---|---|---|---|---|---|---|
| | r | r | P | r | p | r | p | r | r | p | r | p | r | p | r | p | r | p |
| **Proptosis bone** | **latorb°** | | | **medorb°** | | | | **horizontal orbital°** | | | **infraorbslope°** | | | | **latorb-MSP°** | | | |
| distance Mlo-Mlo | | -0.26 | 0.028 | 0.43 | 0.001 | 0.35 | 0.003 | | | | | | -0.31 | 0.009 | 0.47 | < 0.001 | 0.39 | < 0.001 |
| mG-CorparaS | | | | | | -0.25 | 0.039 | | | | | | -0.32 | 0.007 | -0.40 | 0.004 | -0.37 | 0.002 |
| Hertel-values | | | | | | | | | | | | | -0.25 | 0.036 | | | 0.38 | 0.001 |
| Ga-CorparamidApex | | | | | | -0.24 | 0.044 | | | | | | -0.28 | 0.017 | -0.30 | 0.030 | -0.29 | 0.014 |
| mG-MSP | | | | 0.51 | < 0.001 | 0.30 | 0.011 | | | | | | | | 0.42 | 0.002 | 0.39 | < 0.001 |
| Fms-Aperture | | | | 0.29 | 0.037 | | | | | | | | | | 0.39 | 0.005 | 0.48 | < 0.001 |
| **Orbit-skull base position** | | | | | | | | | | | | | | | | | | |
| mMlo-CorparaBa | | | | | | -0.25 | 0.036 | | | | | | -0.31 | 0.009 | -0.58 | < 0.001 | -0.45 | < 0.001 |
| post interorbit width | | -0.25 | 0.037 | | | 0.32 | 0.007 | | | | | | | | | | | |
| ant interorbit width | | | | 0.91 | < 0.001 | 0.90 | < 0.001 | | | | 0.36 | 0.009 | | | 0.34 | 0.014 | 0.33 | 0.005 |
| AO-Cor | | | | | | | | | -0.24 | 0.040 | | | | | | | | |
| Supra-Cor | | | | | | | | | | | | | -0.29 | 0.014 | -0.30 | 0.034 | | |
| Or-Cor | | | | | | | | | -0.29 | 0.016 | | | | | -0.31 | 0.026 | | |
| Mlo-Cor | | | | | | | | | | | | | | | -0.42 | 0.002 | -0.31 | 0.009 |
| Fms-MSP | | -0.25 | 0.038 | 0.51 | < 0.001 | 0.25 | 0.035 | | | | | | -0.28 | 0.019 | 0.58 | < 0.001 | 0.48 | < 0.001 |
| mA-Cor | | | | | | | | | | | | | -0.25 | 0.034 | | | | |
| Da-Cor | | | | | | | | | | | | | -0.28 | 0.020 | | | | |
| OrST-Cor | | | | | | | | | | | | | -0.30 | 0.012 | | | | |
| **Proptosis soft tissue** | | | | | | | | | | | | | | | | | | |
| Ga-CorparaExo | | | | | | | | | | | | | | | | | 0.28 | 0.017 |
| Lidmid-AO | | | | | | | | | | | | | -0.28 | 0.020 | | | | |

Similar results were found for EO and non-EO CT scans regarding the horizontal globe position. A more lateral position was correlated with a larger orbit and wider and higher orbital aperture, larger orbital depth and a retropositioned lateral orbital rim. Concerning Hertel measures (i.e. relative exophthalmos in relation to the orbital aperture and *Nasion*), more correlations were found in the EO group. Generally, a larger orbit was associated with a more anterior globe position. Regarding the position of the orbital rim, larger Hertel values were found in a more retral positioned lateral rim (compared to the orbital aperture midpoint, the medial and superior borders) and a more retral inferior rim (compared to the superior rim).

Concerning orbital angles, most correlations were found for angles between lateral orbital wall and *midsagittal plane*. A larger angle between the lateral wall and the midsagittal plane was correlated to less proptosis, a more retral lateral orbital rim and a more lateral globe in both groups. A larger *infraorbital slope angle* (i.e. a more caudal inferior rim) was associated with less proptosis and a more retral orbital rim position in the non-EO group. *Medorb angle* and *latorb angle* were associated with changes inherent to their definitions (e.g. a smaller *medorb angle* implies a smaller distance between left and right *Dakryon* and thus a smaller anterior interorbital width).

## Discussion

In the discussion of our findings, three aspects had to be considered: First, our method utilizing 3-D cephalometry, then the results regarding the comparison between patients with and without EO and lastly, sexual dimorphism and the influence of anatomy on globe position.

As 3-D cephalometry has already been proven to be reliable regarding intra- and interobserver reliability [16, 17], our study did not include such measurements. 3-D

cephalometry can be performed with an intraobserver landmark selection accuracy of 0.5 to 1.9 mm [18]. We addressed this issue by basing our cephalometric analysis on well-replicable landmark points belonging to Bookstein types 1 and 2 [19].

Further limitations in accuracy lie in the CT slice thickness, which we addressed by excluding CTs with a slice thickness larger than 1.5 mm and the positioning of landmarks. Although the latter is relatively reliable (as mentioned above), it is not without inaccuracy as Smektała et al. [18] found in their systematic review of the experimental and clinical assessement of three-dimensional cephalometry. Thus the use of cross-hairs and the possibility to combine 2-D CT images with 3-D reconstructions for landmark placement as implemented in our investigation should improve the accuracy of our investigation. For further control, several variables were included that did not primarily serve for our analysis and which are well documented like *SNA angle* and *BaSN angle*. Moreover, some of the study parameters are well documented like orbital volume in non-pathologic groups. Here all variables lay within given normative ranges (*SNA angle*, *BaSN angle*; Tab. 7 [20-22]. The same applies to orbital volume.

Table 7: Results for distances in comparison with data from previous studies. Figures in **bold*** were statistically significant differences.

Mean Orbit Volume is given in ml, Mean Orbit Surface in cm², all further parameters are in mm.

| | All Groups | | Male | | Female | | EO | Non-EO |
|---|---|---|---|---|---|---|---|---|
| | Study | Literature | Study | Literature | Study | Literature | Study | Study |
| **Orbit Volume ($cm^3$)** | 29.6 (± 2.6) | 25 – 28.9[13,28-30] | **30.6*** (± 2.6) | 26.8[32] – 29.6[33] | **28.6*** (± 2.9) | 23.2[34] – 26.5[32] | 29.9 (± 3.2) | 29.4 (± 2.7) |
| **Orbit Surface ($cm^2$)** | 55.5 (± 3.7) | | **56.7*** (± 3.1) | | **54.3*** (± 3.6) | | 56 (± 3.8) | 55 (± 3.3) |
| | | | | | | | | |
| **Orbit width** | 41.8 (± 2.1) | 39.8[35] – 41.3[36] | **42.2*** (± 1.6) | 39.8[37] – 44.2[38] | **41.2*** (± 2.2) | 36.9[37] – 42.1[37] | 42.1 (± 1.9) | 41.5 (± 2.1) |
| **Orbit height 2 (Inf-SupM)** | 31.4 (± 2.1) | 33.4[35] – 36.6[28] | 31.4 (± 2.1) | 32.4[10] – 35.9[39] | 31.4 (± 2) | 31.8[10] – 35.4[39] | 31.5 (± 2.1) | 31.4 (± 2.1) |
| **Orbit height (Or-SupF)** | 41.9 (± 2.6) | | 41.7 (± 2.4) | | 42.1 (± 2.5) | | **42.7*** (± 2.2) | **41.4*** (± 2.5) |
| **Orbit lenght medial** | 44.1 (± 3.1) | 43[40] – 46.9[41] | **44.7*** (± 3) | 43.6[40] – 48.6[38] | **43.4*** (± 2.7) | 42.4[40] – 46.2[38] | 44.2 (± 2.8) | 44 (± 3) |
| **Orbit lenght lateral** | 47.5 (± 2.5) | 43.8[40] – 47.2[36] | **48.1*** (± 2.3) | 44.3[40] – 50[38] | **46.8*** (± 2) | 43.3[40] – 48.3[38] | 47.8 (± 2.1) | 47.2 (± 2.4) |
| **Apex – Coronal plane** | 37.5 (± 3.8) | | **38.2*** (± 3.7) | | **36.8*** (± 3.6) | | 37.1 (± 3.7) | 37.8 (± 3.6) |
| **SupM – Coronal plane** | 85 (± 5.2) | | **87*** (± 4.9) | | **82.9*** (± 4.5) | | 84.2 (± 4.7) | 85.6 (± 5.4) |
| **Orbitale – Coronal plane** | 75.7 (± 4.5) | | **77.1*** (± 4.3) | | **74.2*** (± 4.1) | | 74.9 (± 4) | 76.3 (± 4.6) |
| **Supraorb.(Soft Tissue) – Cor** | 94.3 (± 5.7) | | **95.8*** (± 5.4) | | **90.4*** (± 5.8) | | 94.8 (± 4.9) | 93.9 (± 6.1) |
| **Orbit depth (mA-AO)** | 44.5 (± 2.7) | 42[42] | **45.2*** (± 2.6) | 43[36] – 45.2[43] | **43.8*** (± 2.3) | 40.5[34, 36] – 42.8[43] | 44.8 (± 2.4) | 44.3 (± 2.6) |
| **Orbit length superior** | 54 (± 3) | 50.5[36] | **54.9*** (± 2.7) | 51.8[16] – 53.9[38] | **53.1*** (± 2.8) | 51.5[38] – 51.7[16] | 54.1 (± 3.1) | 54 (± 2.8) |
| **Orbit length inferior** | 49.2 (± 3) | 49.6[44] | **50*** (± 2.6) | 47[16] – 50.5[44] | **48.3*** (± 2.8) | 46.9[16] – 48.7[44] | 49.1 (± 3.2) | 49.2 (± 2.6) |
| **Orbit depht (Aof-mA)** | 42 (± 2.7) | | **42.7*** (± 2.4) | | **41.3*** (± 2.5) | | 42 (± 2.7) | 42.1 (± 2.5) |
| **Orbit depth anterior** | 15.5 (± 2.8) | | 15.8 (± 2.8) | | 15.2 (± 2.7) | | 15.8 (± 2.6) | 15.3 (± 2.9) |
| **Infraorbital depth** | 80.4 (± 4.6) | | **81.9*** (± 4.4) | | **78.9*** (± 4.2) | | 79.8 (± 4.5) | 80.9 (± 4.5) |

| | | | | | | | | |
|---|---|---|---|---|---|---|---|---|
| **Sphenoid depth** | 9.5 (± 3) | | 9.2 (± 3) | | 9.7 (± 3) | | **10.3*** (± 2.6) | **8.8*** (± 3) |
| **Ant. sphenoid depth (Mlo-Ast)** | 18.9 (± 2.7) | | **19.6*** (± 2.6) | | **18*** (± 2.4) | | **17.9** (± 2.4) | **19.6*** (± 2.5) |

Generally, the orbital anatomy was highly symmetrical with an average side difference in distances of 0.3 mm and an average side difference in angles of 0.6° which is clinically insignificant and lies within the accuracy range of landmark placement.

This observation is in line with previous evaluations of orbital symmetry that have led to the use of virtual mirroring in the preoperative planning of orbital and midface reconstructive surgery in fractures of the orbit and midface and in navigated surgery [12, 23, 24]. Even if the use of the unaffected side's anatomy as a model for the reconstruction of the affected side is not without limitations [25] it is still the most widely used procedure which is supported by our findings.

Concerning anatomical differences between EO and non-EO patients, Baujat et al. [8] found a slightly smaller lateral orbital angle (corresponding with the angle between lateral orbital wall and midsagittal plane in this investigation) and a smaller interorbital distance in patients with EO compared to non-EO patients with exorbitism. Even if they did not compare EO to non-exophthalmos non-EO CT scans and did not evaluate sex influences, the authors concluded that although minor anatomical differences might exist, orbital anatomy is, at most, of little relevance to the etiology of EO [8].

In our study, 15 parameters including 2 angles showed statistically significant differences between patients with and without EO in women and only one in men. Why men presented less anatomical differences remains speculative. A possible explanation is that the increase of

soft tissue within the orbit might lead to bony resorption and remodeling leading to larger dimensions. The combination of smaller orbit size and potential differences in sex-specific bone structure [26] could thus lead to more remodeling in thinner bone in women (i.e. especially the medial wall) which would explain the larger orbit volume and smaller interorbit distances (Tab.3) in women with EO. Thus a possible explanation of the found anatomical differences in women is, that these are not a cause of EO but rather a consequence. This corresponds to the mechanism of auto-decompression in EO concerning the medial and inferior orbital walls [27]. The lateral orbital angle *(latorbline – midsagittal plane)* which Baujat et al. [8] described as higher in patients with EO did not differ in our study. Instead, it was significantly larger in women. As no analysis of the influence of sex was included in the research of Baujat et al. [8], we suppose that the difference concerning this angle was rather a display of sexual dimorphism than a parameter relevant to the etiology of EO. Still, we agree with Baujat's et al. [8] finding that the orbital anatomy is generally very similar in patients with and without EO, even though there might be minor differences in orbital geometry. Thus, orbital anatomy is unlikely to have a meaningful role in the etiology or extent of EO.

Besides comparing EO and non-EO CT scans which showed little difference we matched our findings with previously published studies (Tab. 7 & 8).

Our data concerning orbital anatomy is generally in line with the results of previous evaluations of orbital anatomy. Concerning distances and angles, only studies with similar definitions of the particular parameters were included in our comparison. Matching with data on EO investigations is difficult, as no data for most of our variables exists to our knowledge. Regarding non-EO data, all comparable measurements matched our findings with little differences concerning distances [13, 16, 28 – 44] or angles [8, 20 – 22, 28, 32, 45 – 47].

Table 8: Results for angles in comparison with data from previous studies. Figures in **bold*** were statistically significant in our study.

| | All Groups | | Male | | Female | | EO | | Non-EO | |
|---|---|---|---|---|---|---|---|---|---|---|
| | **Study** | **Literature** | **Study** | **Literature** | **Study** | **Literature** | **Study** | **Literature** | **Study** | **Literature** |
| **SNA (°)** | 80.2 (± 3.8) | | 80 (± 4.2) | 80.8[20] – 82.5[20] | 80.4 (± 3.6) | 80.5[21] – 82.8[20] | 80.3 (± 3.9 | | 80.1 (± 3.8) | |
| **BaSN (°)** | 130 (± 5.3) | 127.1[22] – 133.5[22] | 131 (± 6.1) | | 129.3 (± 4.6) | | 129.4 (± 5 | | 130.6 (± 5.6) | |
| **Latorbangle (°)** | 86.4 (± 2.4) | 90 | 86.4 (± 2.5) | | 86.4 (± 2.4) | | 86.2 (± 2.7) | | 86.7 (± 2) | |
| **Medorbangle (°)** | 20.1 (± 2.7) | | 19.8 (± 2.6) | | 20.2 (± 2.8) | | 20 (± 2.5) | | 20.2 (± 2.9) | |
| **Globe axis (°)** | 25.1 (± 2.7) | | 24.4 (± 2.3) | | 25.8 (± 2.3) | | 25.1 (± 2.4) | | 25.2 (± 2.4) | |
| **Orbit angle horizontal (°)** | 50.8 (± 3.3) | 45[45] – 53.6[28] | 51.5 (± 3.9) | 47.9[46] – 53.6[32] | 50.2 (± 2.7) | 48.1[46] – 50.8[32] | 50.8 (± 3) | | 50.7 (± 3.6) | |
| **Orbit angle horizontal (Sella) (°)** | 35.4 (± 2.1) | | 35.7 (± 1.8) | | 35.2 (± 1.9) | | 35.1 (± 1.8) | | 35.8 (± 2) | |
| **Infarorbital slope (°)** | 7.2 (± 9.2) | | 6.5 (± 6.2) | | 7.7 (± 9.8) | | 6.2 (± 8.3) | | 8.3 (± 8.6) | |
| **Lateral orbital slope (°)** | 21.8 (± 3.6) | | 22 (± 3.2) | | 21.6 (± 3.7) | | 22 (± 3.6) | | 21.6 (± 3.3) | |
| **Orbithordiam – Coronal plane (°)** | 17 (± 3.6) | | 17.1 (± 3.1) | | 17.0 (± 3.5) | | 16.8 (± 3.6) | | 17.3 (± 3) | |
| **Latorbline – Midsagittal plane (°)** | 45 (± 6.3) | 45[47] | 45.5 (± 2.1) | | 45.3 (± 2.2) | | 45 (± 2.4) | 42[8] | 45.7 (± 1.8) | 40[8] |

Regarding sexual dimorphism in orbital anatomy, previous studies have shown statistically significant differences in orbital volume with men having larger orbit volumes [16, 30, 31] as well as in orbital morphology [11, 16, 39, 43].

In our study (Tab. 4), 41 variables were significantly different, most being larger in men with an average difference of 1 - 2 mm, leading to a 2 ml larger orbit volume. Only orbital proptosis measured in relation to the orbital aperture was larger in women. Additionally, statistically significant differences existed in 1 angle parameter. Regarding orbital volume measurements, several methods have been suggested [13, 48, 49] which will lead to somewhat different results as the anterior confinement is calculated by way of different approaches (which in many reports are proprietary algorithms of the respective authors and not publicly available). Thus the results yielded by our approach lie at the upper range. Deveci et al. found an average of 28.4 ml [29], Regensburg et al. 28.9 ml in Caucasian men [31], whereas the findings of Graillon et al. and Wagner et al. lay at over 26 ml [13, 30]. Nevertheless, the sex difference of 2 ml was similar [16, 30].

Whether the differences between male and female orbits would be mostly due to scaling or in shape was not addressed in this study, but will be an issue of further investigations.

The last issue concerns the relation of globe position to the anatomy of the orbit, especially the orbital aperture. Regarding influential factors on exophthalmos, several studies have shown that globe protrusion diminishes with age while the orbital aperture size increases [50 - 53]. Furthermore Body Mass Index (BMI) seems to play a role [54] as increased BMI is reported to correlate with globe protrusion. Interestingly, no study could be found by the authors that tried to correlate bony anatomic measures with globe position as performed in this study. Only one investigation by Shin et al. [55] measured distance relations of the orbital rim to the globe in a Korean study group, but did not provide further statistical analysis, whereas Kim et al. [56] reported on the significance of orbital rim to globe relation for forensic

reconstructions. Thus our report is the first known to the authors which correlates orbital rim configuration and orbit measures to globe position (Fig. 3). Our findings regarding globe position and anatomy can be differentiated in two aspects. First, internal bony measurements and second, importance of the bony aperture configuration. Looking at intraorbital measurements, the finding that longer orbits correlate with longer distances between orbital apex and globe may seem trivial, but they are not. By now, no correlation of bony size and size of the soft tissue contents is known to the authors. The only known investigation to the authors is the study of Li et al. [46], who investigated the correlation of orbital volume and orbital wall length to ocular protrusion. Similarly to our investigation a positive correlation of orbital volume and a more foreward globe position was identified. However, the authors did not specify how exophthalmos was measured. From the given values it can be inferred, that they used a calculated Hertel value and not the true distance of the globe to the orbital apex as performed in our investigation.

Fig. 3: Visualization of anatomic parameters defining globe position measurements. An increase  of the displayed distances (as stated in red arrows) anterior to the orbital aperture will lead to increased Hertel values, whereas an increase of distances within the orbit will lead to larger distances of the globe to the orbital apex. Analysis planes (parallel planes to *Cor through Da*, *SupM,* and *AO* are visualized as blue lines).

The opposite result, i.e. that a larger orbit might favour a more retropositioned globe or a “shallow” orbit would statistically be correlated to more exophthalmos would also have been understandable. That this was not the case in this investigation is a truly novel finding. Secondly, our results demonstrate the importance of the orbital rim configuration on visually perceived exophthalmos, even if the “true” position of the globe to the orbital apex is unchanged. In a clinical setting, both Hertel and Naugle exophthalmometers cannot be used

for this task as they rely on horizontal bony structures (lateral rim & *Nasion*) or supra- and infraorbital rim to define globe position. Using an elaborate CT analysis, we could show the influence of all orbital rims. Even if it might seem self-explanatory that a retruded lateral orbital rim would increase Hertel values without the change of the globe, this has not been reported on before. Our results showed correlation coefficients in the non-EO group of 0.57 – 0.69 which can be interpreted as moderate to strong.

From a clinical viewpoint, this is of importance, as retruded orbital rims mimick exophthalmos due to other reasons and can be a cause for surgical intervention. Thus in non-EO patients, polyethylene implants are used to augment a retruded orbital rim [57] or in EO treatment, the lateral rim may be advanced-rotated foreward [15, 58]. Utilizing our 3-D cephalometric analysis, it is possible to distinguish between real exophthalmos (i.e. increased distance globe to orbital apex) and pseudoexophthalmos were the globe remains unaltered but the orbital rim is deficient. This knowledge might be important in choosing the appropriate surgical therapy if an intervention is planned.

As EO is a relatively rare disease, the number of patients which can be included is limited. If it was possible to include more patients, the statistical analysis could be even more convincing. Still, our analysis included more patients than the majority of evaluations of orbital anatomy (Baujat et al., 2006: 105 patients; Weaver et al., 2010: 39 patients; Moon et al., 2020: 24 patients; Ji et al., 2010: 64 patients) [8, 10, 11, 16] and should thus be more statistically robust.

As our research included both a larger number of patients as well as the largest number of evaluated anatomical parameters using 3-D cephalometry, our results should not be seen as redundant but as a confirmation of supposed anatomical conditions.

Out of 23 previous evaluations referenced (Tab. 7 and 8), positioning of 3-D landmarks on

3-D reconstructions of the bony orbit was carried out in 5 cases according to the respective methods chapters. Due to the reproductibility of 3-D landmarks, we suppose that methods of orbital analysis utilizing 3-D landmarks on 3-D objects are superior to alternative approaches e.g. measuring directly in 2-D or on 3-D reconstructions regarding precision and comparability.

Thus, we hope to provide useful data in both surgical planning and further research on orbital anatomy and EO e.g. the distance to the sphenoid trigone and its anterior posterior dimensions, a surgical parameter in decompression surgery discussed in Cruz et al. 2021. [59].

All patients included in this research were of Caucasian ethnicity. As differences in orbital anatomy between ethnicities exist [11, 35], it could be of interest to examine whether our observations are valid in other ethnicities as well.

## Conclusions

In this study the relevance of orbital anatomy concerning EO was investigated utilizing 3-D cephalometry in a Caucasian population. As few statistically significant differences between the groups with and without EO were found, anatomy seems to be of minor importance at best concerning the onset of EO. Regarding sex, our investigation confirmed known aspects and added new 3-D data. Sex-related anatomical differences regarding orbital size and the configuration of the orbital aperture (e.g. the more prominent supraorbital rim) may have an impact on the appearance of EO patients and trigger therapeutical demands. This leads to the last aspect where this investigation introduced the interdependence of relative (i.e.to the orbital aperture) and absolute (to the orbital apex) globe position on anatomical parameters. Besides investigating the relevance of anatomy, the anatomical values may be helpful in surgical decision making and treatment planning.

## Declaration of competing interests

The authors declare that they have no conflicts of interest.

### Ethics statement

Our study was approved by the Leipzig University ethics commission (No. 285-14-25082014) and performed in accordance with the Declaration of Helsinki.

## References


1. Gould DJ, Roth FS, Soparkar CNS. The Diagnosis and Treatment of Thyroid-Associated Ophthalmopathy. Aesth Plast Surg. 2012 Jun;36(3):638-48.
2. Bartalena L, Baldeschi L, Dickinson A, Eckstein A, Kendall-Taylor, P, Marcocci C, et al. European Group on Graves' Orbitopathy (EUGOGO): Consensus statement of the European Group on Graves' orbitopathy (EUGOGO) on management of GO. Eur J Endocrinol. 2008 Mar;158(3):273-85.
3. Eckstein A, Berchner-Pfannschmidt U, Führer D, Esser J. Update endokrine Orbitopathie. Ophthalmologe. 2013 Nov;110(11):1079-96. German.
4. Bartalena L, Baldeschi L, Boboridis K, Eckstein A, Kahaly G J, Marcocci C, et al. The 2016 European Thyroid Association/European Group on Graves' Orbitopathy Guidelines for the Management of Graves' Orbitopathy. Eur Thyroid J. 2016 Mar;5(1):9-26.
5. Dollinger J. Die Druckentlastung der Augenhöhle durch Entfernung der äußeren Orbitawand bei hochgradigem Exophthalmos (Morbus Basedowii) und konsekutiver Hauterkrankung. Dtsch Med Wochenschr. 1911 Oct;37(41): 1888-1890. German.
6. Wolfe SA. Modified three-wall orbital expansion to correct persistent exopthalmos or exorbitism. Plast Reconstr Surg. 1979 Oct;64(4):448-55.

7. González-Garcia R, Sastre-Pérez J, Rodriguez-Campo Fj, Naval-Gías L, Monje F. C-modified osteotomy for bilateral advancement of the orbital rim in Gravesorbitopathy: a technical note. Int J Oral Maxillofac Surg. 2008 Sep;37(9):853-7.

8. Baujat B, Krastinova D, Bach CA, Coquille F, Chabolle F. Orbital Morphology in Exophthalmos and Exorbitism. Plast Reconstr Surg. 2006 Feb;117(2):542-50; discussion 551-2.

9. Rajabi MT, Borjian MA , Hosseini SS, Rajabi MA, Hosseinzadeh F, Mohammadi SS. Orbital radiologic parameters of non-syndromic exorbitism patients in comparison with normal population. J Curr Ophthalmol. 2019 Aug 30;31(4):432-437.

10. Weaver AA, Loftis KL, Tan JC, Duma SM, Stitzel JD. CT Based Three-Dimensional Measurement of Orbit and Eye Anthropometry. Invest Ophthalmol Vis Sci. 2010 Oct;51(10):4892-7.

11. Moon SJ, Lee WJ, Roh TS, Baek W. Sex-related and racial variations in orbital floor anatomy. Arch Craniofac Surg. 2020 Aug;21(4):219-224.

12. Jansen J, Dubois L, Schreuers R, Gooris PJJ, Maal TJJ, Beenen L F, et al Should Virtual Mirroring Be Used in the Preoperative Planning of an Orbital Reconstruction. J Oral Maxillofac Surg. 2018 Feb;76(2):380-387.

13. Wagner MEH, Gellrich N, Friese K, Becker M, Wolter F, Lichtenstein JT, et al. Model-based segmentation in orbital volume measurement with cone beam computed tomography and evaluation against current concepts. Int J Comput Assist Radiol Surg. 2016 Jan;11(1):1-9.

14. Hierl T, Huempfner-Hierl H, Kruber D, Gaebler T, Hemprich A, Wollny G. Requirements for a universal image analysis tool in dentistry and oral and maxillofacial surgery. In: Daskalaki A, editor. Dental computing and applications. Advanced techniques for clinical dentistry. Hershey, London: IGI Global; 2009. p. 79-89.

15. Krause M, Huempfner-Hierl H, Kruber D, Sterker I, Hierl T. Calculation of resected

orbital wall areas in the treatment of endocrine orbitopathy. J Craniomaxillofac Surg. 2017 Apr;45(4):485-490.

16. Ji Y, Qian Z, Dong Y, Zhou H, Fan X. Quantitative morphometry of the orbit in Chinese adults based on a three-dimensional reconstruction method. J. Anat. 2010 Nov;217(5):501-6.

17. Olszweski R, Zech F, Cosnard G, Nicolas V, Macq B, Reychler H. Three-dimensional computed tomography cephalometric craniofacial analysis: experimental validation in vitro. Int J Oral Maxillofac Surg. 2007 Sep;36(9):828-33.

18. Smektała T, Jędrzejewski M, Szyndel J, Sporniak-Tutak K, Olszewski R. Experimental and clinical assessment of three-dimensional cephalometry: A systematic review. J Craniomaxillofac Surg. 2014 Dec;42(8):1795-801.

19. Bookstein FL. A Course in Morphometrics for Biologists. Cambridge: Cambridge University Press; 2018.

20. Park S, Yu H, Kim K, Lee K, Baik H. A proposal for a new analysis of craniofacial morphology by 3-dimensional computed tomography. Am J Orthod Dentofacial Orthop. 2006 May;129(5):600.e23-34.

21. Gateno J, Xia JJ, Teichgraeber JF. New 3-dimensional cephalometric analysis for orthognathic surgery. Oral Maxillofac Surg. 2011 Mar;69(3):606-22.

22. Gong A, Ji L, Wang Z, Li Y, Hu F, Li Q, et al. Cranial base characteristics in anterioposterior malocclusions: A meta-analysis. Angle Orthod. 2016 Jul;86(4):668-80.

23. Pierrefeu A, Terzic A, Volz A, Courvoisier D, Scolozzi P. How Accurate Is the Treatment of Midfacial Fractures by a Specific Navigation System Integrating „Mirroring“ Computational Planning? Beyond Mere Average Difference Analysis. J Oral Maxillofac Surg. 2015 Feb;73(2):315.e1-315.e10.

24. Gui H, Yang H, Zhang S, Shen SG, Ye M, Schmelzeisen R: Mirroring Tool: The Simplest Computer-Aided Simulation Technology?. J Craniofac Surg. 2015

Oct;26(7):2115-9.

25. Sozzi D, Gibelli D, Canzi G, Tagliaferri A, Monticelli L, Cappella A, et al. Assessing the precision of posttraumatic orbital reconstruction through „mirror“ orbital superimposition: A novel approach for testing the anatomical accuracy. J Craniomaxillofac Surg. 2018 Aug;46(8):1258-1262.

26. Barkaoui A, Kahla RB, Merzouki T. Age and gender effects on bone mass density variation: finite elements simulation. Biomech Model Mechanobiol. 2017 Apr;16(2):521-535

27. Vaidya A, Lee PAL, Kitaguchi Y, Kakizaki H, Takahashi Y. Spontaneous orbital decompression in thyroid eye disease: new measurement methods and its influential factors. Graefes Arch Clin Exp Opthalmol. 2020 Oct;258(10):2321-2329.

28. Ramieri G, Spada MC, Bianchi SD, Berrone S. Dimensions and volumes of the orbit and orbital fat in posttraumatic enophthalmos. Dentomaxillofac Radiol. 2000 Sep;29(5):302-11.

29. Deveci M, Oztürk S, Sengezer M, Pabuşcu Y. Measurement of orbital volume by a 3-dimensional software program: an experimental study. J Oral Maxillofac Surg. 2000 Jun;58(6):645-8.

30.Graillon N, Boulze C, Adalin P, Loundou P, Guyot L. Use of 3D orbital reconstruction in the assessment of orbital sexual dimorphism and its pathological consequences. J Stomatol Oral Maxillofac Surg. 2017 Feb;118(1):29-34.

31. Regensburg NI, Wiersinga WM, van Velthoven MEJ, Berendschot TTJM, Zonneveld FW, Baldeschi L, et al. Age and gender-specific reference values of orbital fat and muscle volumes in Caucasians. Br J Ophthalmol. 2011 Dec;95(12):1660-3.

32.Kamer L, Noser H, Schramm A, Hammer B, Kirsch E. Anatomy-Based Surgical Concepts for Individualized Orbital Decompression Surgery in Graves Orbitopathy. I. Orbital Size and Geometry. Ophthalmic Plast Reconstr Surg. Sep-Oct 2010;26(5):348-

52.

33. Ching JA, Ford JM, Decker SJ. Aging of the Adult Bony Orbit. J Craniofac Surg. 2020 Jun;31(4):1082-1085.

34. Kamer L, Noser H, Schramm A, Hammer B, Kirsch E: A step towards individualized, anatomy-based surgical concepts for orbital decompression in Graves' orbitopathy. Orbit. 2009;28(4):237-40.

35. Masters MP. Modern variation and evolutionary change in the hominin eye orbit [dissertation]. Columbus (OH): The Ohio State University; 2008.

36. Lang J, von Lanz T, Wachsmuth W. Praktische Anatomie. Kopf- Gehirn- und Augenschädel. Berlin, Heidelberg, New York: Springer-Verlag; 1979

37. Lepich T, Dąbek J, Piechota M, Bajor G, Aniszewski L, Markowski J. Digital analysis of the orbit using the non-referring method. Arch Med Sci. 2014 Feb 24;10(1):182-90.

38. Pereira AM, Antunes AA, Soriano EP, Mara Rodrigues BH, Santos Pereira VB, Porto GG. Orbital cavity evaluation in a Brazilian population. J Oral Maxillofac Radiol. Jan-Apr 2019;7(1):1-5.

39. Guyomarc'h P, Dutailly B, Couture C, Coqueugniot H. Anatomical Placement of the Human Eyeball in the Orbit – Validation Using CT Scans of Living Adults and Prediction for Facial Approximation. J Forensic Sci. 2012 Sep;57(5):1271-5.

40. Skorek, Szurowska E, Raczyńska D, Szmuda T, Stodulski D. Orbital size measurement based on computed tomography imaging for surgical safety. Folia Morphol. 2014;73(3):314-320.

41. Chon B, Zhang KR, Hwang CJ, Perry JD. Longitudinal Changes in Adult Bony Orbital Volume. Ophthalmic Plast Reconstr Surg. May/Jun 2020;36(3):243-246.

42. Borumandi F, Hammer B, Noser H, Kamer L. Classification of orbital morphology for decompression surgery in Graves' orbitopathy: two-dimensional versus three-dimensional orbital parameters. Br J Ophthalmol. 2013 May;97(5):659-62.

43. Nitek S, Bakoń L, Sharifi M, Rysz M, Chmielik L P, Sadowska-Krawczenko I: Morphometry of the Orbit in East-European Population Based on Three-Dimensional CT Reconstruction. Advances in Anatomy. Volume 2015, https://doi.org/10.1155/2015/101438

44. Kang H, Han JJ, Oh H, Kook M, Jung S, Park H. Anatomical Studies of the Orbital Cavitiy Using Three-Dimensional Computed Tomography. J. Craniofac Surg. 2016 Sep;27(6):1583-8.

45. René C. Update on orbital anatomy. Eye (Lond). 2006 Oct;20(10):1119-29.

46. Li Y, Su Y, Song X, Zhou H, Fan X. What is the Main Potential Factor Influencing Ocular Protrusion? Med Sci Monit. 2017 Jan 5;23:57-64.

47. Wilkinson MJ. Anatomy of the human orbit. Operative Techniques in Otorhinolaryngology. 2018;*29*(4):186-192.

48. Jansen J, Schreurs R, Dubois L, Maal TJJ, Gooris PJJ, Becking AG. Orbital volume analysis: validation of a semi-automatic software segmentation method. Int J Comput Assist Radiol Surg. 2016 Jan;11(1):11-8.

49. Sentucq C, Schlund M, Bouet B, Garms M, Ferri J, Jacques T, et al. Overview of tools for the measurement of the orbital volume and their applications to orbital surgery. J Plast Reconstr Aesthet Surg. 2021 Mar;74(3):581-591.

50. Ahmadi H, Shams PN, Davies NP, Joshi N, Kelly MH. Age-related changes in the normal sagittal relationship between globe and orbit. J Plast Reconstr Aesthet Surg. 2007;60(3):246-50.

51. Beden Ü, Özarslan Y, Öztürk HE, Sönmez, B,Erkan D, Öge I. Exophthalmometry values of Turkish adult population and the effect of age, sex, refractive status, and Hertel base values on Hertel readings. Eu J Opthalmol. Mar-Apr 2008;18(2):165-71.

52. Shieh AC, Blandford AD, Hwang CJ, Perry JD. Age-related Changes in Globe Position. Ophthalmic Plast Reconstr Surg. Mar/Apr 2019;35(2):155-158.

53. Shaw RB, Katzel EB, Koltz PF, Yaremchuk MJ, Girotto JA, Kajn DM, et al. Aging of the Facial Skeleton: Aesthetic Implications and Rejuvenation Strategies. Plast Reconstr Surg. 2011 Jan;127(1):374-383.

54. Schmidt P, Kempin R, Langner S, Beule A, Kindler S, Koppe T, et al. Association of anthropometric markers with globe position: A population-based MRI study. PLoS One. 2019 Feb 7;14(2):e0211817.

55. Shin KJ, Lee SH, Koh KS, Song WC. Three-dimensional and topographic relationships between the orbital margins with reference to assessment of eyeball protrusion. Anat Cell Biol. 2017 Mar;50(1):41-47.

56. Kim SR, Lee KM, Cho JH, Hwang HS. Three-dimensional prediction of the human eyeball and canthi for craniofacial reconstruction using cone-beam computed tomography. Forensic Sci Int. 2016 Apr;261:164.e1-8.

57. Goldberg RA, Soroudi AE, McCann JD. Treatment of prominent eyes with orbital rim onlay implants: four-year experience. Ophthalmic Plast Reconstr Surg. 2003 Jan;19(1):38-45

58. Krause M, Kamal M, Halama D, Hierl T, Sterker I, Zimmerer R, et al. Eyes wide shut: necessity and effect of adjunctive procedures after decompression surgery in patients with endocrine orbitopathy. Head Face Med. 2021 Sep 15;17(1):41.

59. Cruz AAV, Equiterio BSN, Cunha BSA, Caetano FB, Souza RL. Deep lateral orbital decompression for Graves orbitopathy: a systematic review. Int Ophthalmol. May;41(5):1929-1947.

## CRediT author statement

**Konstantin Volker Hierl:** Data curation, Investigation, Visualization, Writing.

**Matthias Krause**: Additional measurements, Supervision.

**Daniel Kruber**: Programming of FAT Software.

**Ina Sterker***:* Conceptualization of the study, Supervision.

Figure 1

Click here to access/download;Figure;Fig 1.tif

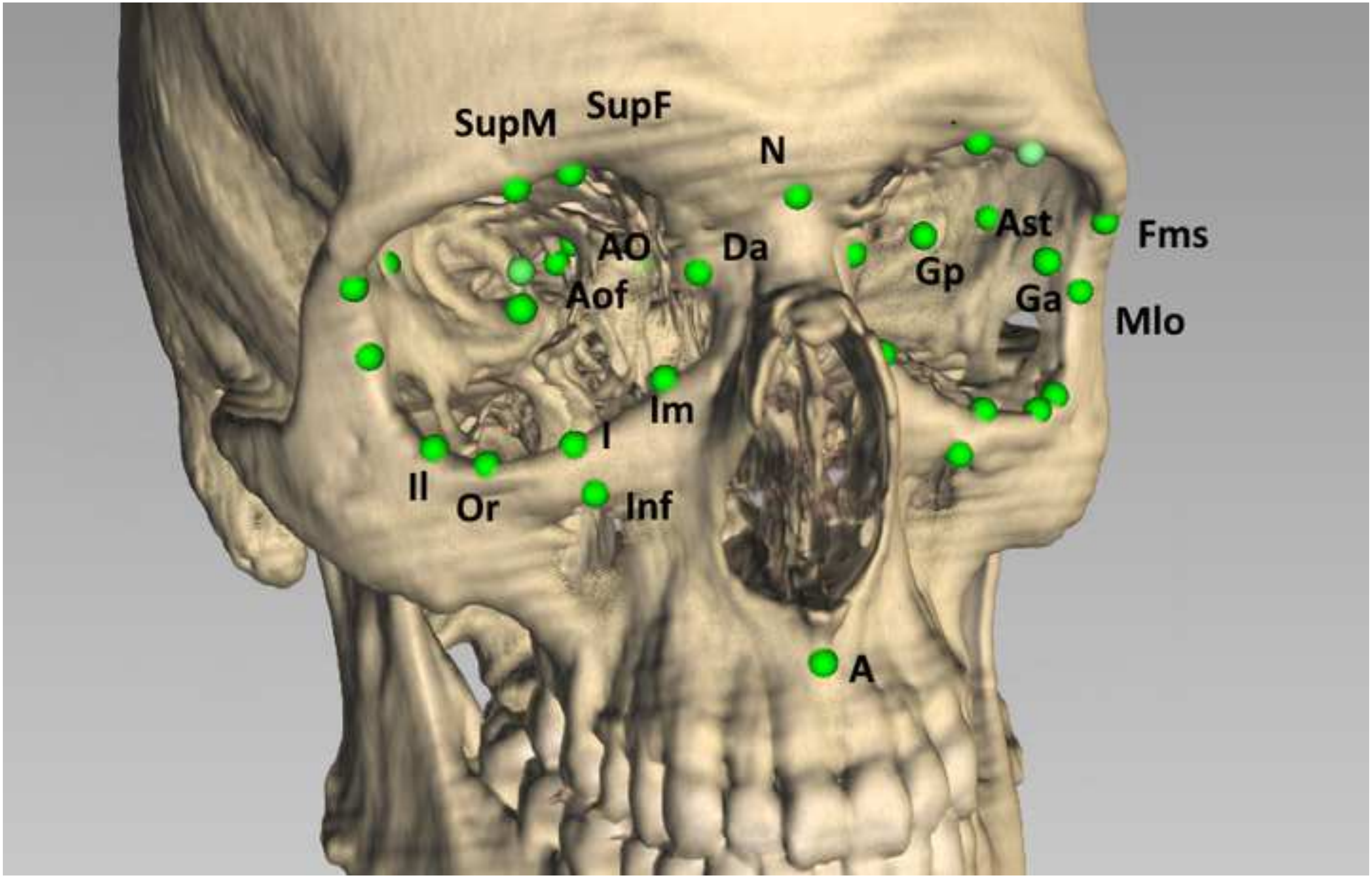

Figure 2

Click here to access/download;Figure;Fig 2.tif

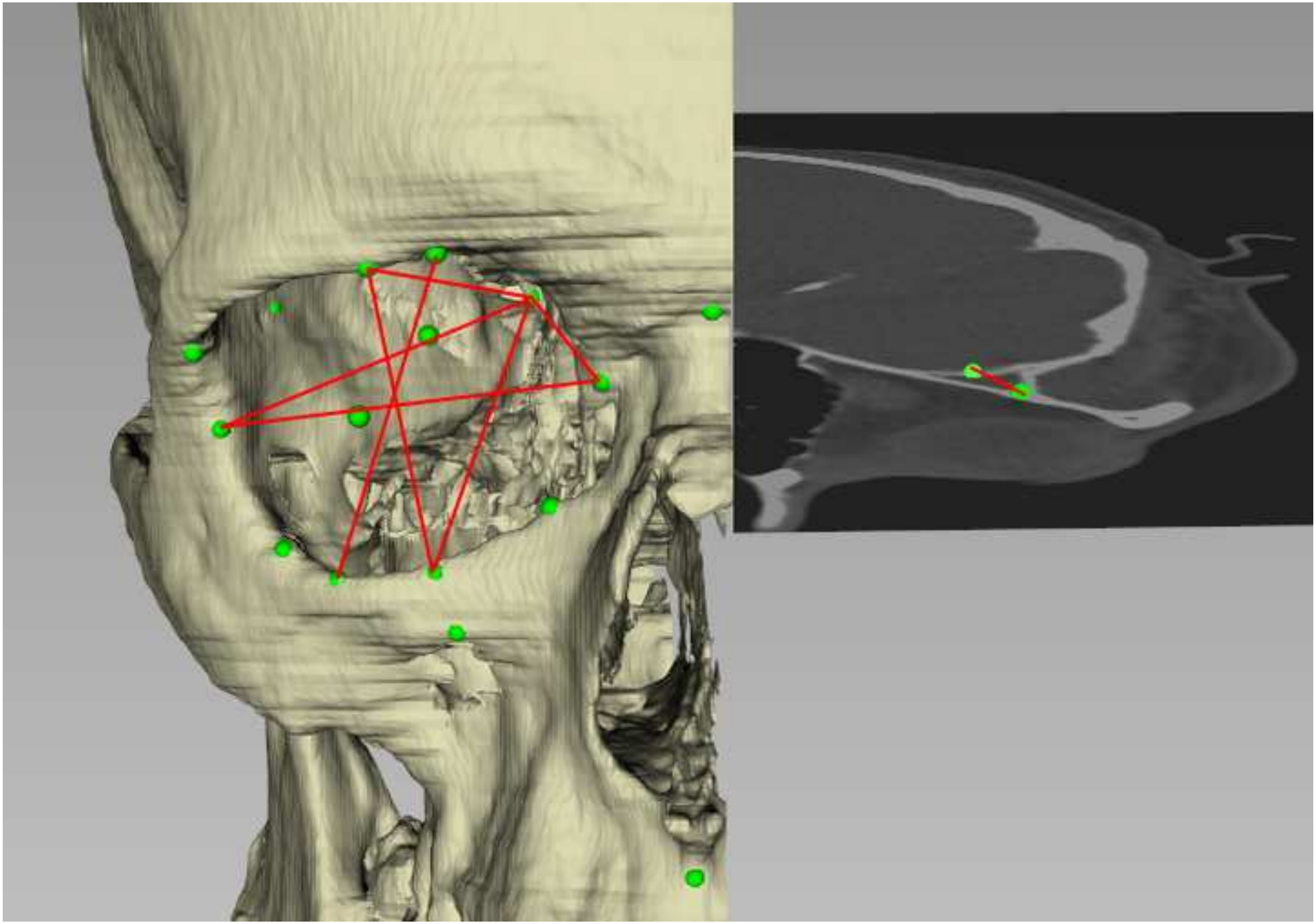

Figure 3

Click here to access/download;Figure;Fig 3.tif

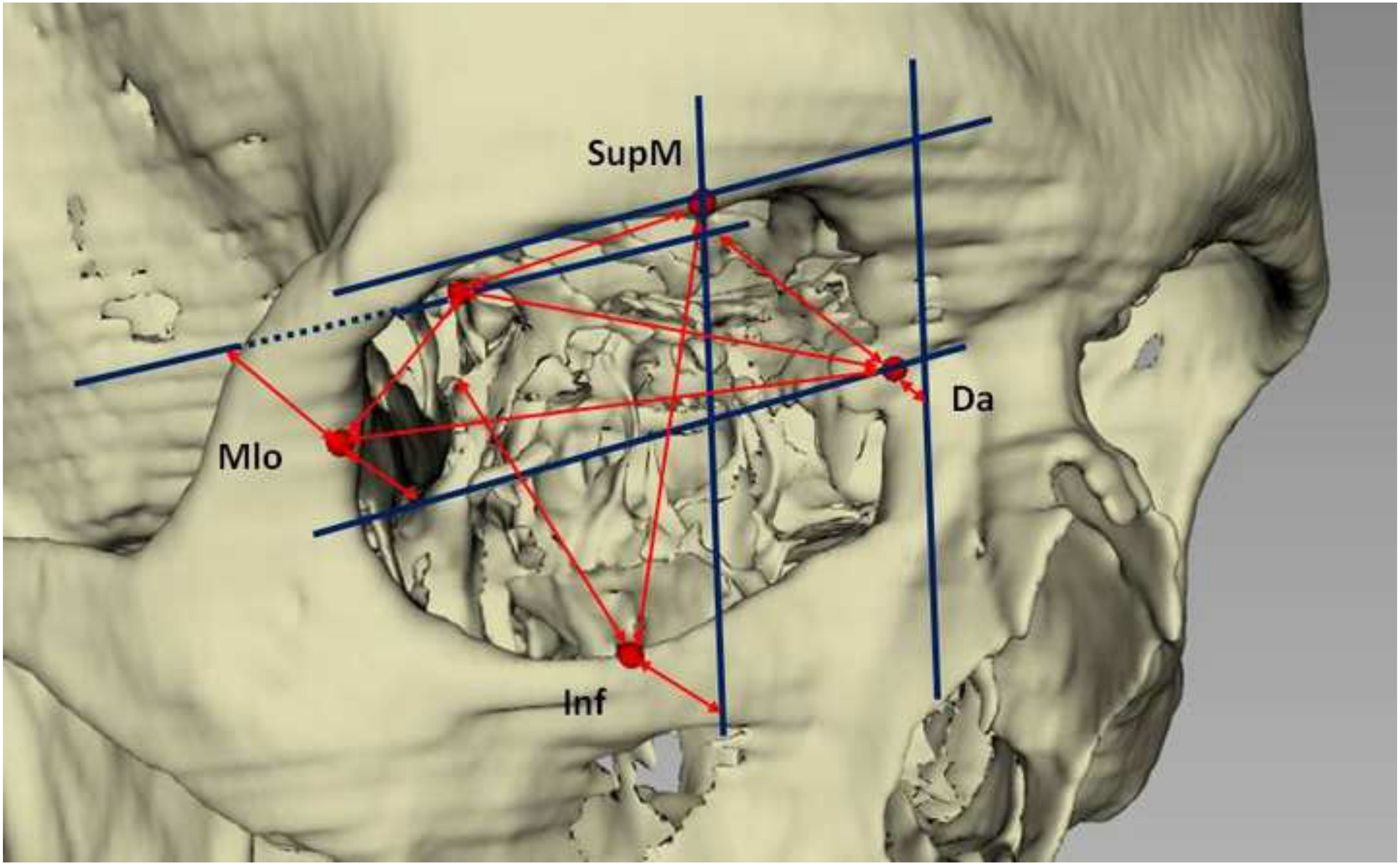

Additional data

Click here to access/download

**Supporting Information**

Data Plos one.xls
