# Supplementary material for: 3-D cephalometry of the the orbit regarding endocrine orbitopathy, exophthalmos, and sex
Source: PLoS One. 2022 Mar 11;17(3):e0265324. doi: 10.1371/journal.pone.0265324 (PMC8916626; doi:10.1371/journal.pone.0265324)
Supplement: Attachment — Submitted filename: Revision notes to journal.docx [file pone.0265324.s003.docx]

Dear Mr Amgain, dear reviewers,

Thank you very much for your comments to improve the quality of the submitted paper draft.

In the following chapters, all remarks will be covered and the text changes marked appropriately in the paper and in this document.

**Remarks by the editor:**

1. *Please ensure that your manuscript meets PLOS ONE's style requirements, including those for file naming. The PLOS ONE style templates can be found at*
   [*https://journals.plos.org/plosone/s/file?id=wjVg/PLOSOne_formatting_sample_main_body.pdf*](https://deref-gmx.net/mail/client/X9RQVttdVls/dereferrer/?redirectUrl=https%3A%2F%2Fjournals.plos.org%2Fplosone%2Fs%2Ffile%3Fid%3DwjVg%2FPLOSOne_formatting_sample_main_body.pdf) *and* 
   [*https://journals.plos.org/plosone/s/file?id=ba62/PLOSOne_formatting_sample_title_authors_affiliations.pdf*](https://deref-gmx.net/mail/client/Psng0f32Rw8/dereferrer/?redirectUrl=https%3A%2F%2Fjournals.plos.org%2Fplosone%2Fs%2Ffile%3Fid%3Dba62%2FPLOSOne_formatting_sample_title_authors_affiliations.pdf)

This has been done.

2. *Please provide additional details regarding participant consent. In the ethics statement in the Methods and online submission information, please ensure that you have specified (1) whether consent was informed and (2) what type you obtained (for instance, written or verbal, and if verbal, how it was documented and witnessed). If your study included minors, state whether you obtained consent from parents or guardians. If the need for consent was waived by the ethics committee, please include this information.*

*If you are reporting a retrospective study of medical records or archived samples, please ensure that you have discussed whether all data were fully anonymized before you accessed them and/or whether the IRB or ethics committee waived the requirement for informed consent. If patients provided informed written consent to have data from their medical records used in research, please include this information.*

This study has been a retrospective investigation on medical records. The local University ethics commission waived the requirement of informed consent as pseudonymization of all data was performed. Thus the following sentence was included on p. 10 at the end of the Materials and methods section (highlighted in green):

This retrospective study was approved by the ~~local~~ Leipzig University ethics commission (No. 285-14-25082014) and followed the Declaration of Helsinki on medical protocols and ethics. As pseudonymization of the CT data was performed, the requirement of informed consent was waived by the ethics committee.

**Reviewer’s Comments to the Author**

Please use the space provided to explain your answers to the questions above. You may also include additional comments for the author, including concerns about dual publication, research ethics, or publication ethics. (Please upload your review as an attachment if it exceeds 20,000 characters)

**Reviewer #1:** The author’s work is appreciated but it would have been better if they incorporate these suggestions to make this manuscript even better.

***1. Please remove the short form (EO) from the title as full form is already mentioned, if possible****.*

The short form (EO) was removed from the title.

***2. In abstract section, please do not start the sentence with number rather start with words like- ‘two’ instead of ‘2’. Please make the conclusion part concise and short.***

2 was changed to as required:

…and without EO. Two out of 12 angles showed a statistically ….

The conclusion was shortened made more focused. The changes were (added: light blue, deleted words: ~~red~~):

~~There appears to be~~ In this study little difference in orbital anatomy between patients with and without EO was found…. Thus, orbital anatomy ~~is likely to be of minor relevance in the etiology of EO but~~ may influence therapy regarding timing and surgical procedures as ~~orbital rim anatomy~~ it affects exophthalmos. ~~Sex-based differences were confirmed.~~

3. ***Title in the beginning and in the main manuscript is different. Please clarify****.*

As the title fulfills the word count requirements of the short title, the short title was changed to the full title.

***4. Please mention the objective of this study at the end of ‘introduction part’ after it’s rationale.***

The objectives of our investigation have now been summarized at the end of the introduction. The changes are:

… ~~The purpose of our study was to evaluate orbital anatomy in patients with and without EO investigating the relevance of orbital anatomy in the etiology of EO and exophthalmos – one of its major clinical features – using a new approach of three-dimensional cephalometric measurement.~~ ~~In previous investigations~~ Utilizing measurements in CT-scans, Baujat et al. and Rajabi et al. [8, 9] did not find ~~such~~ significant anatomical differences in EO patients besides slight differences in the lateral orbital angle (angle between the midsagittal plane and the lateral orbital wall) or interorbital distance. These studies, however, were limited by several factors like the use of axial CT slices for 2-D measurements instead of 3-D cephalometry [8, 9]. Baujat et al. compared two patient groups with exophthalmos (EO and non-EO) [8] whereas Rajabi et al. evaluated a small group~~s~~ of non-EO patients [9]. The number of observed parameters was limited in both evaluations. Moreover, the influence of sex was not evaluated.

Thus, the purpose of our study was to evaluate orbital anatomy in patients with and without EO investigating the relevance of orbital anatomy in the etiology of EO and exophthalmos – one of its major clinical features – using a new approach of three-dimensional cephalometric measurement.

***5. Out of 123 samples, 71 were having no known pathology, doesn’t it raise the ethical concern for radiation exposure while doing CT? Please defend this.***

The control CTs without anatomical discernable pathology had been acquired because of the following reasons: search for foci in the head & neck region due to general illnesses (but none found), search for anatomical causes of neurologic disorders (none found), CT scans in preparation of oral surgery/oral maxillofacial surgery which didn´t cause anatomical alterations with regard to this investigation (e.g. wisdom teeth removal)

The following sentence was added in Materials and methods section:

…group. The CT scans without anatomical discernable pathology (reference group) had been acquired in search for foci in the head & neck region due to general illnesses, in search for anatomical causes of neurologic disorders, and in preparation of oral surgery or oral and maxillofacial surgery not associated with anatomical alterations relevant to this investigation. Besides…

***6. Please mention your own findings only in the result section.***

The results regarding symmetry which had been repeated in the discussion were deleted.

The text was changed to:

…~~Generally, the orbital anatomy was highly symmetrical with an average side difference in distances of 0.3 mm and an average side difference in angles of 0.6°~~ Regarding symmetry, the differences in this investigation were ~~which is~~ clinically…

***7. In discussion, please try not to repeat the result findings again if it's not applicable for discussion with other literatures and also you can remove the reference table and figures from the discussion as it’s already there in the results.***

Both tables in the discussion section have been changed to only display reference data. Repetitions of our results were removed from the discussion section.

***8. There is inadequate discussion on the interpretation and implication of study findings. It would have been better if they have included more comparable studies on discussion.***

Within the discussion section, some aspects have now been treated more in depth.

This includes methodology:

- 3D cepalometry per se (p. 16-17)

and results:

- 3D symmetry (p. 18-19)

- 3D anatomy (p. 20-21)

- sex differences (p. 23-24)

- importance of orbital rim morphology (p. 24-26)

- importance for surgical treatment (p. 27)

More literature was included in the discussion (see references marked in blue)

***9. Please correct some grammatical error in the manuscript.***

Grammar errors have been deleted and style was improved.

***10. Please write the conclusion part in short, clear and concise.***

The conclusion was changed to be more concise and clear.

**Reviewer #2: Portions of the results section have been placed in the methods section. The method section basically focus on the research design, how the study was conducted and how the reader who wants to do the similar study can reproduce the method in this paper**

The materials and methods chapter was checked to include no results. Only data on the patient and control group was provided. Furthermore, only the lists of the utilized landmarks and the measured distances and angles have been presented. Concerning data presentation in the discussion (see reviewer 1/6 & 7), this has be changed.
