# Supplementary material for: 3-D cephalometry of the the orbit regarding endocrine orbitopathy, exophthalmos, and sex
Source: PLoS One. 2022 Mar 11;17(3):e0265324. doi: 10.1371/journal.pone.0265324 (PMC8916626; doi:10.1371/journal.pone.0265324)
Supplement: Attachment — Submitted filename: Covering Letter References to table 1-8.docx [file pone.0265324.s004.docx]

**Revision to New Manuscript: PLOS ONE**

**3-D cephalometry of the the orbit regarding endocrine orbitopathy, exophthalmos, and sex**

Dear Mr. Tating Baluloy,

The references to Table 1-8 and Figure 1-3 in the text were revised.

Kind regards,

Konstantin Volker Hierl
